# Genetic abrogation of immune checkpoints in antigen-specific cytotoxic T-lymphocyte as a potential alternative to blockade immunotherapy

**DOI:** 10.1038/s41598-018-23803-7

**Published:** 2018-04-03

**Authors:** Chi Zhang, Yanchun Peng, Philip Hublitz, Haokang Zhang, Tao Dong

**Affiliations:** 1 MRC Human Immunology Unit, Weatherall Institute of Molecular Medicine, Oxford University, Oxford, OX3 9DS UK; 2 Center for Translational Immunology, Chinese Academy of Medical Science Oxford Institute, Nuffield Department of Medicine, Oxford University, Oxford, OX3 7BN UK; 3 Genome Engineering Department, Weatherall Institute of Molecular Medicine, Oxford University, Oxford, OX3 9DS UK

## Abstract

T cell function can be compromised during chronic infections or through continuous exposure to tumor antigens by the action of immune checkpoint receptors, such as programmed cell death protein 1 (PD-1). Systemic administration of blocking antibodies against the PD-1 pathway can restore T cell function, and has been approved for the treatment of several malignancies, although there is a risk of adverse immune-related side-effects. We have developed a method for generating gene knockouts in human antigen (Ag)-specific cytotoxic T-Lymphocyte (CTLs) using clustered regularly interspaced short palindromic repeats (CRISPR)/CRISPR-associated protein 9 (Cas9) genome editing. Using this method, we generated several transduced CD4^+^ or CD8^+^ antigen-specific polyclonal CTL lines and clones, and validated gene modifications of the PD-1 gene. We compared these T-cell lines and clones with control groups in the presence of programmed death-ligand 1 (PD-L1) and observed improved effector functions in the PD1-disrupted cell group. Overall, we have developed a versatile tool for functional genomics in human antigen-specific CTL studies. Furthermore, we provide an alternative strategy for current cell-based immunotherapy that will minimize the side effects caused by antibody blockade therapy.

## Introduction

In response to the constant antigenic stimulation caused by chronic viral infections, or cancer cell antigens, cytotoxic T lymphocytes (CTLs) often become “exhausted” with sustained expression of inhibitory receptors and a distinct transcriptional state. In this state, CTLs fail to perform their main function of killing their target cells^1^. T-cell exhaustion is mediated by tissue and microenvironment factors, regulatory cytokines and the signals from the immune checkpoint receptors such as cytotoxic T-lymphocyte-associated protein 4 (CTLA-4), and programmed cell death protein 1 (PD-1). Tumour cells can hijack immune checkpoint pathways as a way to induce immune resistance to CTLs that are specific for tumour antigens^2,3^. The ability to restore these immune responses offers new approaches to treatment, making the modulation of T-cell immunity one of the most exciting areas of cancer research in recent years. Since 2011, a series of antibody therapies that act on the immune checkpoint receptors CTLA-4 and PD-1, and their ligands, have been approved by the FDA and have been relatively successful in treating a range of malignancies^4–6^. Simultaneously, engineering patient autologous T-cells to express chimeric antigen receptors (CARs) using replication-deficient viruses has led to long-term remission of B-cell neoplasms in some leukemia patients^7,8^, but these too may be susceptible to checkpoint inhibition. In light of these developments, immunotherapy is playing an ever greater role in the cancer treatment, alongside the traditional treatments of surgery, radiotherapy and chemotherapy^9^.

CTLs are distinguished from other blood cells by their capacity to directly kill specific target cells using cytolytic molecules^10^. In chronic viral infection or cancer, CTLs recognizes antigenic peptides presented by major histocompatibility complex (MHC) from the target cells and unleash potent killing. However, only a small proportion in the total T-cells pool inside one’s body recognizes a specific viral or cancer antigenic peptide. Therefore, systemic administration of antibodies that interfere with immune checkpoint pathways will act on all T cells and can lead to a breakdown in the discrimination of self and non-self, resulting in the onset of autoimmune disorders^11^. Thus, immune-related adverse events (iRAEs) are frequently observed in patients who receive antibodies that act on immune checkpoints, occurring in up to 90% and 70% of patients that are treated with anti-CTLA4^4^ or anti-PD-1/PD-L1 antibodies^12,13^, respectively. Though steroids can be used to relieve iRAEs, the anti-tumour responses induced by antibody therapies can be compromised by such generalised immune-suppression^11^. Therefore, specific and intrinsic disruption of immune checkpoints in antigen-specific T-cells through genetic targeting may be needed to give a better safety profile for immunotherapy^14^.

Replication-deficient pseudotyped lentiviral vectors are widely used as tools in basic research^15^, as well as for treatment of human diseases such as inherited genetic disorders^16,17^, and, more recently, cancers^18,19^. CRISPR associated protein 9 (Cas9) is an RNA-guided endonuclease, which is widely used as a simple and affordable way to edit mammalian cell genome^20^. There have been several successful studies on engineering primary T-cells using CRISRP/Cas9. Schumann and colleagues delivered pre-assembled sgRNA and Cas9 protein via electroporation into human CD4 primary T cells, resulting site-specific mutations in CXCR4 and PD-1 genes^21^. Su and colleagues mutated the PD-1 gene using plasmid electroporation in the peripheral CD8^+^ T cells of cancer patients or healthy individuals, and showed increased immune responses against cancer antigens^14^.

**Figure 1:**
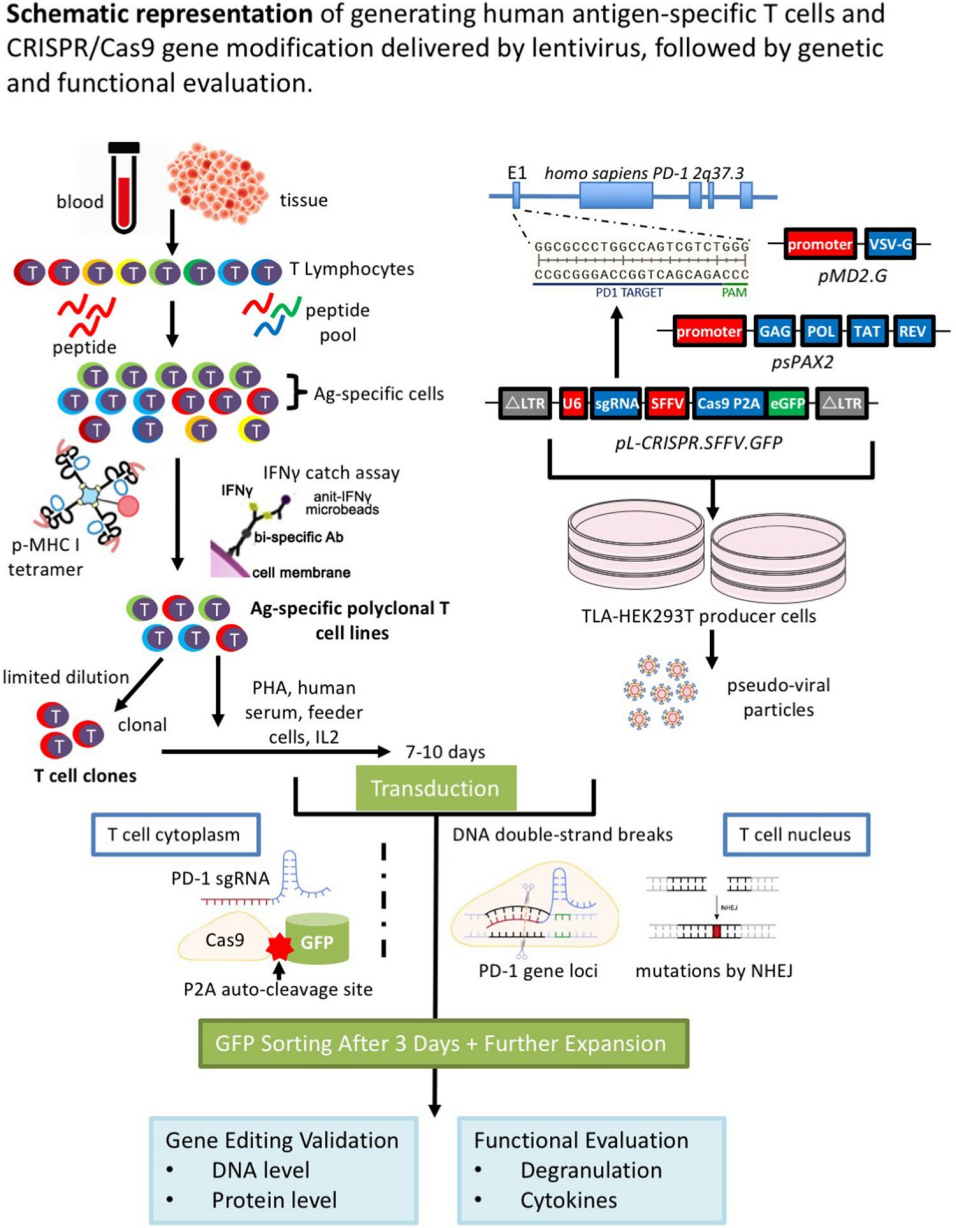
Schematic representation of generating PD-1 KO antigen-specific polyclonal CTL lines. The antigen-specific T cells (both CD4 and CD8) were isolated from patients and cultured *in vitro* using feeder cells plus PHA, and could be further enriched or cloned for functional study. Lentivirus co-expressing Cas9, eGFP and specific sgRNA was used to transduce antigen-specific T cells and knock out (KO) immune checkpoint genes, such as PD-1. The gene KO effect can be examined by checking at the DNA and protein level. Functional studies include cytokine quantifications and CD107a staining can be carried out to evaluate T cell functions.

Here we used CRISPR/Cas9 genome editing together with lentiviral delivery to disrupt PD-1 gene expression in selected human antigen-specific polyclonal CTLs (Fig. 1), a procedure that could confer better activity and a better safety profile for immunotherapy with antigen specific T cells. Herein, we conducted a proof-of-concept study to ascertain the feasibility of knocking-out the PD-1 gene using lentivirus in antigen-specific polyclonal CTLs. Functional improvements of T cell “quality” in the methodology and evaluation of the knock-outs were monitored by cytokine production and degranulation. Our results underscore a potential application of isolating antigen-specific CTLs from patients, *in vitro* gene editing and expansion, and then transfusion back into patients for treatments of cancers and/or chronic infections.

## Results

### Validation of PD-1 sgRNA and lentiviral transfer

**Figure 2:**
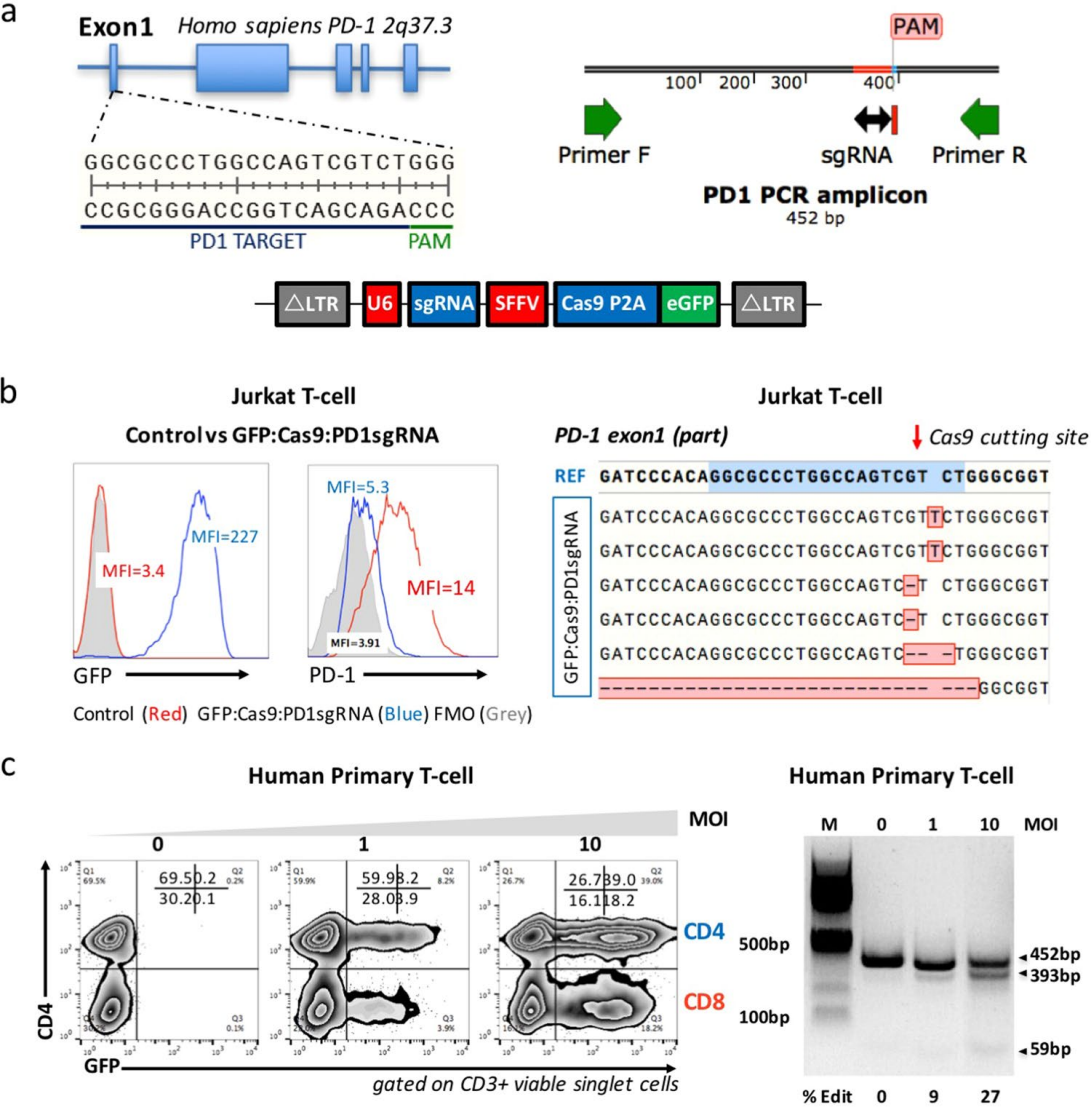
The validation of PD-1 sgRNA/Cas9-mediated PD-1 knock-out via lentivirus in Jurkat T-cells and human primary T-cells. (**a**) Schematic diagram of sgRNAs targeting the PD-1 exon 1 locus (upper left), PCR amplicon (upper right), and the lentiviral backbone (bottom). (**b**) Examples of GFP and PD1 expression in non-transduced (control) and GFP:Cas9:PD1sgRNA transduced Jurkat T-cells. (MOI = 10) Jurkat cells transduced with lentivirus show a significantly lower level of expression of PD1 compared to non-transduced cells. FMO control shown in grey shaded area. Sanger sequencing confirmed the PD-1 down-regulation was due to site-specific mutations caused by sgRNA/Cas9. (**c**) The transduction method was further validated on pre-stimulated human primary T-cells. Viruses with a MOI of 0, 1 and 10 were used to transduce cells, the PCR amplicon were tested for T7E1 cleavage assay. The above experiments have been repeated 3 times with similar results.

The Human *PDCD1* gene is located on Chr2q37.3^22^ and contains 6 exons (Fig. 2a). We used CRISPR/Cas9 to specifically target and disrupt PD-1 in human T cells. Three PD-1 targeting sgRNAs were designed and cloned into pL-CRISPR.SFFV.GFP, which delivers Cas9, GFP and PD-1 targeting sgRNA (Cas9:GFP:PD1sgRNA) simultaneously (Supplementary Fig. 1a). sgRNA#1, targeting PD-1 exon1, was used in the following experiments as it generated the strongest PD-1 down-regulation among the three designed sgRNAs (Supplementary Fig. 1b). The Cas9:GFP:PD1sgRNA virus mediated efficient PD-1 disruption in Jurkat T-cells (Fig. 2b). Genomic DNA harvested from these Jurkat T-cells was checked for the presence of site-specific gene disruptions by Sanger sequencing of the PCR amplicon surrounding the target site (Fig. 2b). Indel mutations were detected within the sgRNA targeting site, indicating successful CRISPR/Cas9-mediated DNA double-strand break formation (Fig. 2b). To further validate the lentiviral delivery method, pre-stimulated human primary T-cells (with anti-CD3/CD28 magnetic beads) were transduced with Cas9:GFP:PD1sgRNA virus at different MOIs, cells were cultured for three days, and the GFP expression monitored by flow cytometry (Fig. 2c). Both CD4^+^ and CD8^+^ T-cells were efficiently transduced, as indicated by GFP expression. T7E1 cleavage assays revealed mismatches within the PCR amplicon (Fig. 2c). These data demonstrate that the selected sgRNA worked effectively with Cas9 to disrupt the PD-1 gene, and the lentiviral transduction method delivered Cas9:GFP:PD1sgRNA into human T-cells.

### Generation of PD-1 KO human antigen-specific polyclonal CTL clones/lines

**Figure 3:**
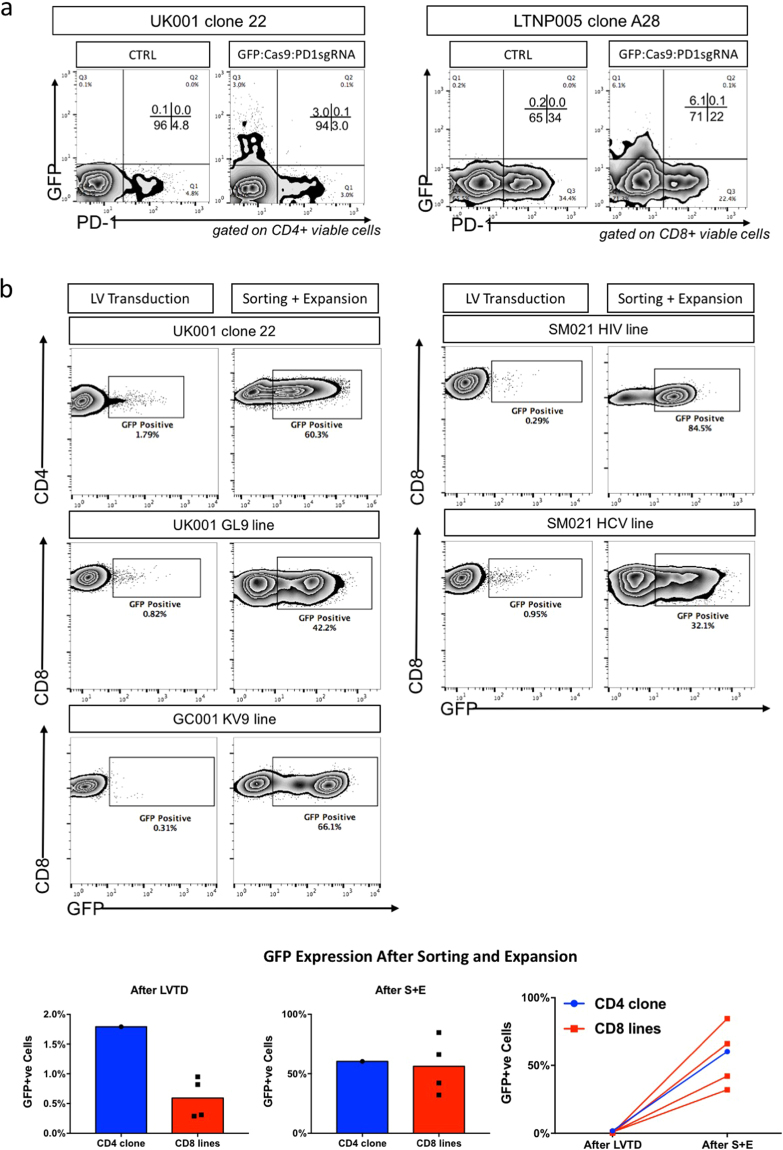
Lentiviral transductions in *in vitro* cultured antigen-specific CTL lines. (**a**) Representative PD1-GFP plots of one CD4 (left) and one CD8 (right) antigen-specific T-cell clone. For each group, the control cells (mock lentiviral transduction) are on the left and the GFP:Cas9:PD1sgRNA transduced cells are on the right. In general, three days after transduction, only a small proportion of virally transduced cells became GFP positive. (**b**) CD4-GFP plot of one CD4 antigen-specific T-cell clone and CD8-GFP plot of five antigen-specific CD8 CTL lines. Lentiviral transduced cells (GFP positive cells, left) could be enriched by GFP sorting and further expanded by feeder cells plus PHA (right). GFP positive cells comprised up to 80% of the cells after one round of sorting plus the expansion procedure. Summaries of GFP expression sorting patterns are plotted at the bottom. LVTD: lentiviral transduction; S + E: sorting and expansion.

Each T-cell clone has a specific rearrangement of its TCR genes and each clone recognizes a specific epitope or antigen. Thus, in many experimental settings, it may be difficult to attribute a certain function to a defined T-cell population by studying the overall population of T-cells^23^. Using an MHC-peptide tetramer, T-cells with TCRs that recognize a certain epitope can be isolated from the peripheral blood T-cell pool and further expanded by feeder cells for detailed study^24^. Previously, we have accumulated a bank of both CD4 and CD8 antigen-specific polyclonal CTL lines and CTL clones. (further cloned from lines). The antigen specificities range from acute infections (such as influenza), chronic infections (such as hepatitis C virus, and HIV-1), and cancers (such as tumour-associated antigen (TAA) specific cells). Lentiviral transductions were performed unitizing those pre-established antigen-specific CTLs (Table 1). After initial viral transduction, the frequency of GFP-positive (and also Cas9-positive, due to co-transcription of the Cas9 and GFP cassettes, which separate via an auto-cleavage site) T-cells in the population was less than 5%, although those cells were also PD-1 negative, possibly indicating successful PD-1 gene disruptions (Fig. 3a). In contrast to the stimulated human primary T-cells (Supplementary Fig. 2a), *in vitro* cultured antigen-specific T-cell clones were refractory to lentiviral transduction. The low efficiency could not be overcome by using polycations, or further CD3/CD28 stimulation (Supplementary Fig. 2b). Thus, another round of fluorescence-activated cell sorting was applied to enrich the GFP-positive cells. After GFP enrichment and further expansion, the frequency of GFP-positive cells within the total population was as high as 80% (Fig. 3b).

**Table 1:** Information of the antigen-specific polyclonal CTL lines or clones used in this study.

CTL ID	HLA Restriction	CD4/CD8 dependency	Specificity
UK001 clone22	DRB1*0901	CD4	Influenza H3 HA_344–354_ RI11
LTNP005 clone A28	B*0801	CD8	HIV Nef_90–97_ FL8
UK001 GL9 line	A*0201	CD8	Influenza M1_58–66_ GL9
SM021 HIV Nef line	Multiple	CD8	HIV Nef
SM021 HCV NS3 line	Multiple	CD8	HCV NS3
270435 KV9 line	A*0201	CD8	Cancer Specific SSX-2_41–49_ KV9

### Efficient Cas9-mediated PD-1 disruption in the primary T-cells of patients

**Figure 4:**
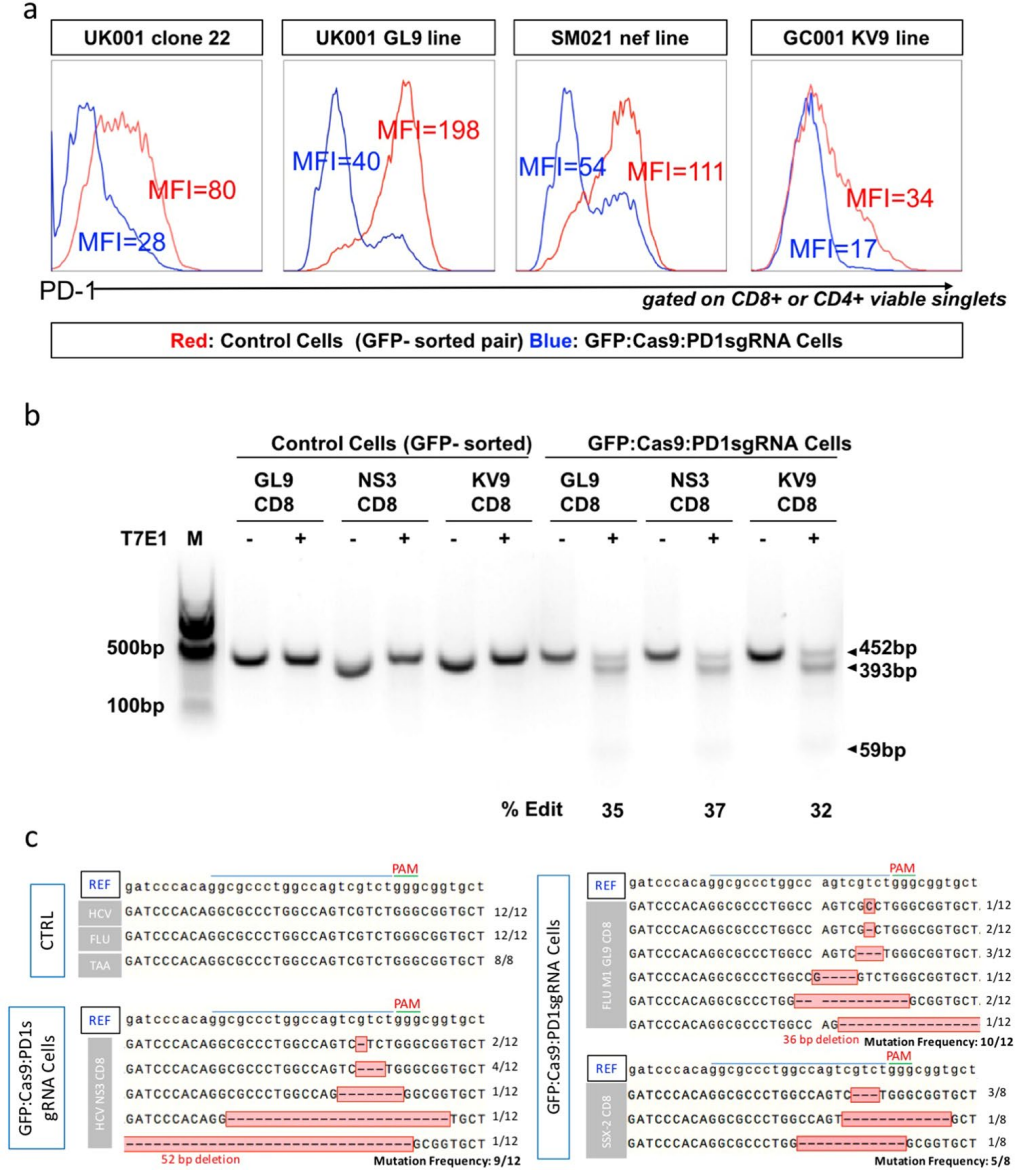
Validation of PD1 KO effect on antigen-specific CTL lines. (**a**) PD1 expression in lentiviral transduced (GFP:Cas9:PD1sgRNA) and non-transduced (control) antigen specific T-cell lines. PD1 expression in T-cells was checked 24 h post anti-CD3/anti-CD28 stimulation. With stimulation, almost all the control cells express PD1, but in the majority of GFP:Cas9:PD1sgRNA cells PD1 expression declines (both in terms of MFI and percentage). (**b**) T7E1 cleavage assays were conducted to reveal the indel mutation within the sgRNA targeting region in the GFP:Cas9:PD1sgRNA cells. Total editing frequency is shown at the bottom. (**c**) Sanger sequencing of the PCR product flanking the sgRNA site. No indel mutations were detected in control cells, in contrast to GFP:Cas9:PD1sgRNA cells where the sgRNA targeting sites were heavily mutated.

PD-1 expression in T-cells is both tightly and dynamically regulated. PD-1 is up-regulated in response to immune challenges and is rapidly down-regulated in acute settings to allow normal immune responses. Its expression remains high in chronic settings leading to an impaired immune response to stimuli^25^. The expression of PD-1 in *in vitro* cultured antigen-specific polyclonal CTL cells is low unless they are stimulated by co-culturing with anti-CD3/CD28 magnetic beads. To further characterize the GFP-sorted and expanded cells, and validate that GFP selection could distinguish PD-1 knock-out cells from the population, four pairs of lentiviral-transduced and GFP-sorted antigen-specific CTL cells were stimulated and their PD-1 expression was determined after 24 hours (Fig. 4a). In each case, cells transduced with PD-1-targeting lentivirus that were GFP positive expressed much lower levels of PD-1 on their cell surface compared to their control.

To validate that PD-1 down-regulation on these antigen-specific T-cells was caused by site-specific mutations genomic DNA was extracted from the three CD8 antigen-specific CTL cell-line pairs, and PCR amplicons of the surrounding region were cloned for sequencing. T7E1 assays and Sanger sequencing of the three control pairs detected no mutations in the region around sgRNA target site. However, a heterogeneous collection of mutations was identified in all three GFP:Cas9:PD1sgRNA transduced pairs (Fig. 4b,c). The detailed PD-1 MFI value and mutation rate for each group are summarised in Table 2. Taken together, these results demonstrate the efficient disruption of the PD-1 gene at both DNA and protein level.

**Table 2:** Summary of PD-1 expression levels on different transduction pairs.

CTL ID	Control Cells	GFP:Cas9:PD1sgRNA Cells				
	PD1 + PCT	PD-1 MFI	PD1 + PCT	PD-1 MFI	T7E1	Sequencing
UK001 clone22	66.5%	79.4	25.9%	27.8	—	—
UK001 GL9 line	87.6%	198	20.8%	39.3	35%	10/12
SM021 HCV NS3 line	71.2%	111	34.3%	54.4	37%	9/12
270435 KV9 line	37.6%	33.6	11.2%	16.9	32%	5/8

PCT: percentage, MFI: median fluorescent intensity.

### Inhibition of T-cell functions by the ligand of PD-1

**Figure 5:**
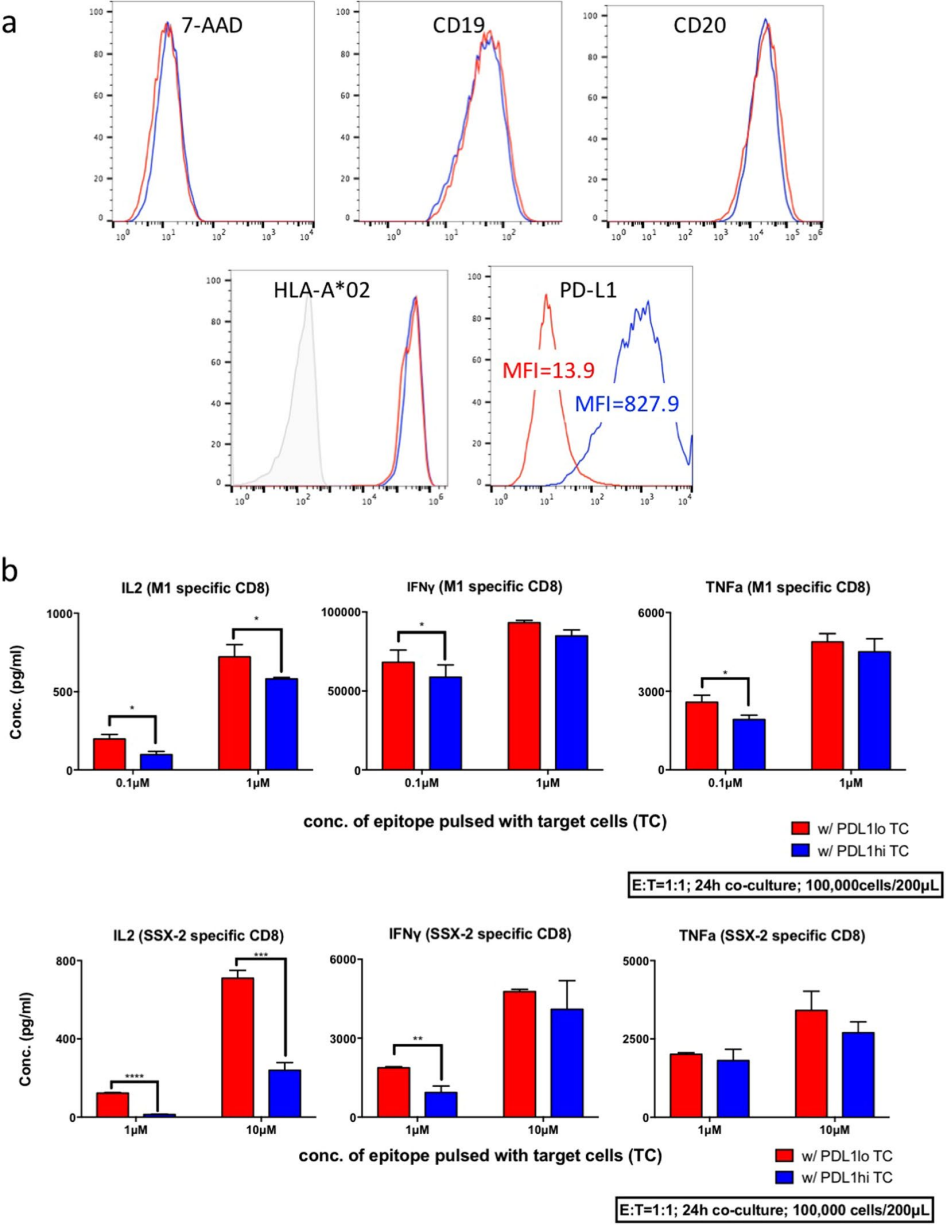
PD-L1 can inhibit T-cell cytokine secretions. (**a**) PD-L1, CD19, CD20, HLA-A*02 and viability in non-transduced and PD-L1 ORF virus transduced HLA-A*0201/ DRB1*0901 BCLs (target cells). (**b**) Influenza A M1-specific and SSX-2 specific CD8 CTL lines were co-cultured with peptide-pulsed PD-L1-lo or PD-L1-hi BCL target cells with corresponding amount of peptide, cells were cultured for 24 h and the supernatants were harvested and subjected to Luminex assay for cytokine quantification. Data shown are mean ± SD of three independent experiments and we depicted a representative out of three experiments yielding similar results. *Indicates p-value < 0.05. **Indicates p-value < 0.01. ***Indicates p-value < 0.001. ****Indicates p-value < 0.0001. p-values were calculated using two-tail unpaired student’s t-test. All experiment has been repeated for three times.

PD-L1 and PD-1 engagement induces T-cell apoptosis^26^ and functional suppression^27^. Under physiological conditions, few normal human tissues express PD-L1. In contrast, PD-L1 protein is highly expressed on the cell surface in many human cancers, as indicated by immunohistochemistry in frozen human tumour sections^28^. To validate that high levels of PD-L1 expression result in the inhibition of antigen-specific CTLs, we overexpressed PD-L1, using our lentivirus expression system, in EBV-transformed HLA-A*02 B lymphoblastoid cell lines (BCLs). Compared to non-transduced BCLs, lentiviral-transduced BCLs show a dramatic increase in PD-L1 protein levels on the cell surface, with no change in expression of either CD19, CD20, HLA-A*02 molecules, nor any change in viability (Fig. 5a).

GL9 (M1_58–66_) is an immunodominant HLA-A*02-restricted CTL epitope in influenza virus^29^. KV9 (SSX-2_41–49_) is a tumour-specific HLA-A*02-restricted epitope within the SSX-2 gene, which is expressed in neoplasms of various histological types^30^. To evaluate PD-L1 inhibition of T-cell functions, supernatant from the co-culture of effector cells (wild-type M1_58–66_- or SSX2_41–49_-specific CTL bulk lines) and target cells (epitope-loaded PD-L1-low (lo) or PD-L1-high (hi) BCLs) was collected for cytokine quantification by Luminex multiplex assay. Among the CTL cytokines, interleukin 2 (IL-2) is important for the proliferation of T lymphocytes^31^; interferon γ (IFNγ) has anti-viral, anti-tumour, and immunoregulatory properties^32–34^; while tumour necrosis factor α (TNFα) is a multi-functional pro-inflammatory cytokine that is involved in the regulation of a wide spectrum of biological processes^35^. By co-culturing antigen-specific CTL cells with PD-L1 low BCL or PD-L1 high BCL (target cells), T-cells showed decreased production of cytokines when target cells presented limiting amounts of peptide and expressed higher levels of PD-L1. IL-2 production was particularly reduced (Fig. 5b, Supplemental Fig. 3).

### Invigoration of T-cells by ablating PD-1 in an immune-suppressive microenvironment

We tested if the loss of function of PD-1 can invigorate T-cell functions in the presence of high levels of PD-L1. PD-L1 inhibition is known to be more effective when lower amounts of antigen are presented to the antigen-specific CTLs (Fig. 4b). Thus, 0.1 μM and 1 μM of epitope was used to stimulate M1-specific CD8 CTLs and SSX2-specific CD8 CTLs, respectively. Lentiviral-transduced CTL pairs (GFP:Cas9:PD1sgRNA and control cells) were co-cultured with PD-L1-hi or -lo HLA-matched target cells (HLA-A*02 BCL) with or without antigen, and the supernatants were collected and analysed.

**Figure 6:**
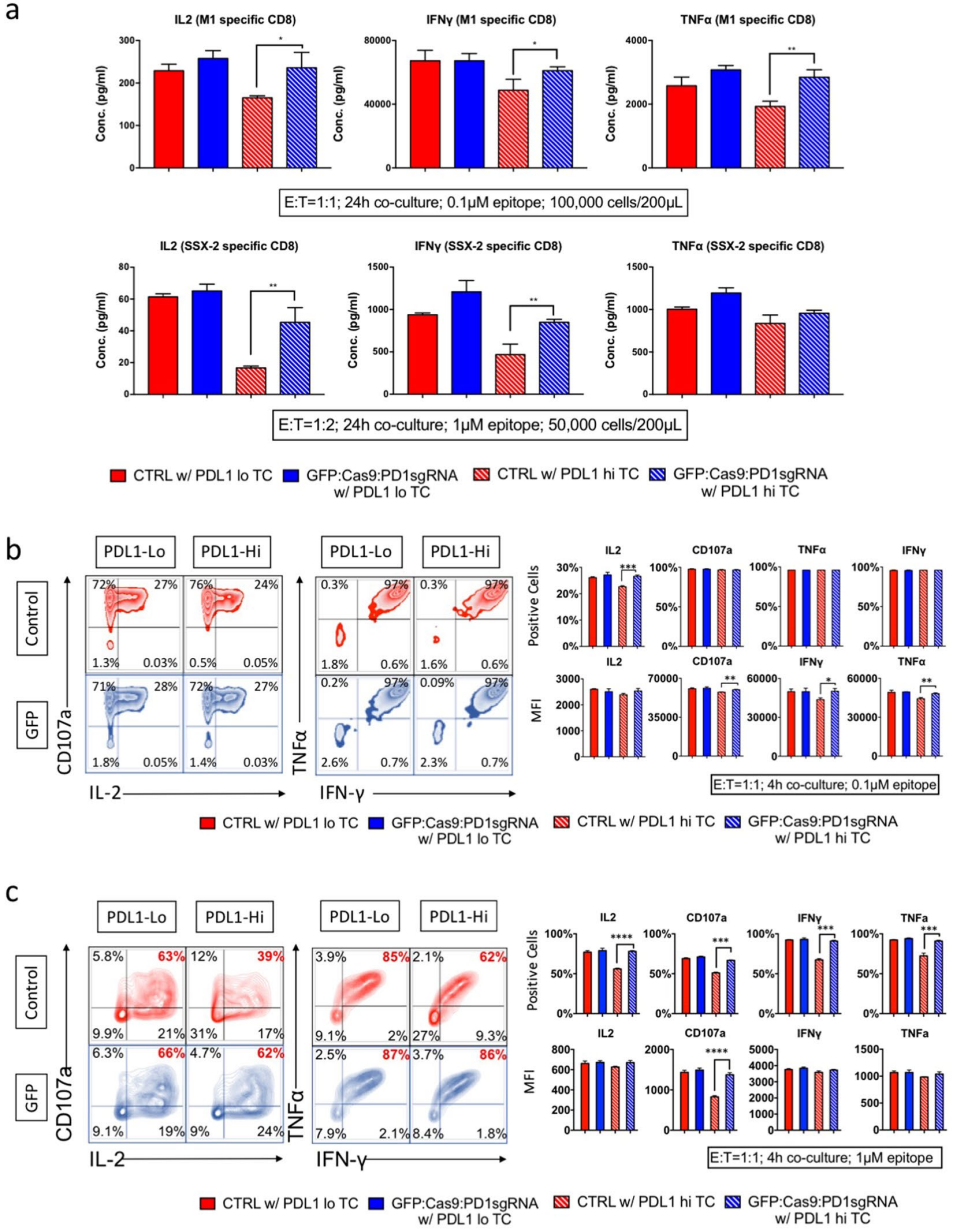
Disrupting PD-1 to release PD-L1 inhibition on T-cells. (**a**) IL2, IFNγ, TNFa cytokine production of effector cells and target cell co-cultures (24 h) were quantified by Luminex assay for M1-specific and SSX-2-specific CD8 CTL lines. Control effector cells co-cultured with PD-L1-hi target cells showed decreased IL2, IFNγ, and TNFα production compared to co-culture with PD-L1-lo target cells, while GFP:Cas9: PD1sgRNA effector cells produced comparable amount of these cytokines with both PD-L1-lo and –hi groups. (**b**,**c**) FACS plot (left) and bar chart (right) for M1-specific CD8 T-cell line pair and H3HA specific CD4 T cell clone 22 pair. Control effector cells co-cultured with PD-L1-hi target cells showed decreased degranulation (indicated by CD107a staining) and cytokine production. similar results. *Indicates p-value < 0.05. **Indicates p-value < 0.01. ***Indicates p-value < 0.001. ****Indicates p-value < 0.0001. p-values were calculated using two-tail unpaired student’s t-test. All experiment has been repeated for three times.

In both antigen-specific CTL lines, GFP:Cas9:PD1sgRNA cells produced comparable levels of IL-2, IFNγ, and TNFα in the presence of high-level or low-level PD-L1, which is in contrast to the control cells (Fig. 6a). In a similar experimental setting, the M1-specific CD8 cells and H3 HA-specific CD4 clone22 cells were co-cultured with the HLA matched target cells in the presence of Golgi transporter blockers, and then stained to determine intercellular level of cytokines, and surface levels of CD107a, which is present in the membranes of cytotoxic granules and is transported onto the cell surface as a result of degranulation^36^, representing T-cell degranulation status. CTLs lentiviral-transduced to ablate the PD-1 gene produced comparable amount of IL2, IFNγ and TNFα with PD-L1-lo target cells but significantly higher amounts of these cytokines with PD-L1-hi target cells. In addition, the GFP:Cas9:PD1sgRNA-transduced T-cells exerted similar level of degranulation with the control T-cells in the PD-L1-lo BCL group, in contrast to the higher expressions in the PD-L1-hi BCL group (Fig. 6b,c).

These findings confirm the ablation of PD-1 on antigen-specific CTL cells can overcome PD-L1 inhibition and allow the immune system to regain the control of the target cells, as indicated by the higher level of degranulation and cytokine production.

## Discussion

The introduction of CAR or TCR transgenic T-cells has revolutionized the field of immunotherapy and provided evidence for the feasibility of T cell-based strategies to treat cancers. However, targeting solid tumours using CAR T-cells remains problematic^37^. Unlike circulating blood, the tumour microenvironment is, in many cases, immunosuppressive; transformed cells and local immune cells express high-levels of the PD-1 ligand. PD-L1 binding to PD-1 results in the phosphorylation of tyrosine on the cytoplasmic tail of PD-1, which recruits SHP2 (SH2-domain containing tyrosine phosphatase 2), which then dephosphorylates PI3K (phosphatidylinositol-4,5-bisphosphate 3-kinase). The inactivation of PI3K dampens downstream Akt signalling, and hampers T-cell activation, proliferation, and survival^38^. One strategy to counter PD-1 inhibition is by the systemic administration of anti-PD1 or anti-PD-L1 blocking antibodies, which has already been approved by the FDA for treating different cancers, although patients suffer the risk of developing autoimmune disorders^12,13^. Combination use of PD-1-blocking antibodies and CAR T-cells in mice has been reported to enhance the anti-tumour efficacy^39^, however, this will make the treatment more costly and carries a higher risk of breaking peripheral immune tolerance.

Thus, autologous tumour-specific T-cells with a loss-of-function mutation in the PD1 gene could provide an alternative cancer treatment. The CRISPR/Cas9 system has been adapted to engineer genome in many species; several studies have reported the successful engineering of the PD-1 gene in primary T-cells using CRISRP/Cas9^14,21^. Nevertheless, those studies engineered the PD-1 gene in primary T-cells without pre-selection/sorting, which, in a similar fashion to antibody therapy, could induce T-cells to target self-antigens. Here, we described the first PD-1 knock out in antigen-specific CTL lines via a lentivirus based method.

The main purpose of this work was to establish a platform to edit genes in antigen-specific cells rather than the total primary T cell population, potentially bypassing any autoimmune responses. This approach, by knocking out immune checkpoint receptor(s), together with the identification of new tumour antigens, could lead to better T-cell-based therapy, especially in those cases where no appropriate antibodies are available for constructing chimeric receptors. Furthermore, PD-1 disruption can be used in combination with other immune checkpoint knock-outs, such as T-cell immunoglobulin and mucin-domain containing-3 (TIM-3) as blocking PD-1 can induce compensatory TIM-3 up-regulation; genes that promote T-cell death, such as FAS could also be knocked out in combination with other immune checkpoint receptors, in order to achieve optimal anti-tumour effect by cytotoxic T cells.

Blockade of the PD-1 pathway is already known to improve effector cell IFNγ production, as well as cytotoxicity, both *in vitro*^40^ and *in vivo*^39^. Here we also demonstrated that the GFP:Cas9:PD1sgRNA-modified antigen-specific CTL cells from patients exhibited enhanced IFNγ production, by co-culturing with the related antigen-loaded target cells. At the same time, we also detected enhanced degranulation potency and production of IL-2. Our observations that PD-L1 inhibition could only be observed in PD-L1-hi groups and not PD-L1-lo group indicate that the improved T-cell functions seen in GFP:Cas9:PD1sgRNA cells in the PD-L1 high environment was indeed due to the CRISPR/Cas9 mediated PD-1 mutations. However, the differences between control and GFP:Cas9:PD1sgRNA-modified antigen-specific CTL cells from patients were shown to be marginal. This could be due to other factors that affect T cell functions, as the contribution of PD-1 inhibitory pathway to T cell functions could also depend on the functional avidity of each T cell line and clone used, cytokine production profile upon stimulation, other inhibitory and co-stimulatory molecules expressed on the cells etc.

Lentiviral viral vectors and Cas9 proteins or peptides are also a concern due to their potential immunogenicity given their non-human origin. *In silico* predictions suggest that the Cas9 protein contains peptide sequences that might bind to many common HLA class I or class II alleles^41^. Therefore, humanizing Cas9 protein is a potential strategy to minimize the immunogenicity of Cas9 peptides^42^. It has been reported that adenoviral delivered Cas9 is immunogenic as demonstrated by the *in vivo*^43^ and Cas9-specific antibodies could be detected *in vivo*^44^. However, these vibrant immune responses are most likely adenovirus-derived as low immunogenicity was observed with hydrodynamic plasmid delivery and AAVs^41^. There are not many immunogenic reports of lentiviral delivered Cas9, however, constantly expressed CRISPR/Cas9 may give rise to undesirable off-target effects. ‘Hit and run’ therefore is a better strategy, such as electroporating ribonucleoprotein (RNP) or plasmids. However, in our initial attempts to electroporate *in vitro* cultured T cell clones, cells underwent intensive cell death and failed to recover (data not shown). Further optimizations are needed for *in vitro* culture of antigen-specific T cell lines. Integrase-deficient lentiviral vector (IDLV) may serve as an alternative tool, as the vector does not integrate into host genome but preserves the broad tropism to different cell types^45^. These additional approaches provide interesting areas for future research into the optimization of this delivery system.

We noticed that the GFP:Cas9:PD1sgRNA cells we have generated proliferate much slower than wild type cells after multiple rounds of expansion, particularly after manipulations such as lentiviral transduction and GFP sorting. This could be *due to extensive manipulations of our T cell lines/clones or could be related to an underappreciated role for PD-1 in the prevention of over-activation-induced dysfunction, as suggested by murine studies*^46^. We evaluated the gene editing effect via both T7EI assay and sanger sequencing. According to Schumann *et al*.^21^, the T7E1assay, a gene editing assessment method that is convenient but could also underestimate editing efficiency, uses denaturation and hybridization of the wild-type and mutant sequences to create a mismatch DNA duplex, which is then digested by T7 endonuclease. However, hybridization of the mismatch duplex may be inefficient, especially when the indel mutation is drastically different from the wild-type sequence, making self-hybridization an energetically more favorable product. Other possibilities include incomplete duplex melting, inefficient cleavage of single-base-pair indels, etc. More genuine editing efficiency is reflected by Sanger sequencing. We also noticed a lower frequency of GFP expressing cells than gene-edited cells than sanger sequencing. Although we don’t have direct evidence, we think it is because the GFP expression was lost in some of PD-1 edited cells. We think it may because some T cells silenced lentiviral genome or lost the viral genome somehow after the genome being edited by Cas9. In addition, PD-1 knock out cells expressed higher TIM-3 than control pairs when stimulated with anti-CD3/CD8 beads (Supplementary Fig. 4). We are currently exploring the possibility of knocking out apoptosis genes as well as other immune checkpoint genes together with PD-1 to make the cells both more sustainable and functionally capable of lysing antigen.

Although we have shown that we are able to knock out PD-1 on antigen specific T cells, sort and expand *in-vitro*. Prior to the use of this method in the clinic there are improvements to be made, in particular the requirement of sorting and limited expansion potential of gene-edited cells. However, the advantage of this method is to prevent the outgrowth of non-antigen-specific or PD-1-positive cells, such as occurred when primary T cell populations were used^14,21^. By sorting and expanding antigen specific cells, PD-1 deleted cells for adoptive transfer even with limited numbers may still be much more effective than using primary T cells without sorting and expansion.

In conclusion, herein we have established a platform for disrupting an immune check-point gene (PD-1) in human antigen-specific polyclonal CTLs. The technique could be widely used and easily applied to other immune checkpoint genes. Our gene editing method is suitable for functional studies of single or combined mutations in immune checkpoint receptors and, more importantly, has the potential to be adapted for clinical applications such as the treatment of infection, autoimmunity, and cancers.

## Methods

### Plasmids and Antibodies

pL-CRISPR.SFFV.GFP(Addgene plasmid # 57827) was a gift from Benjamin Ebert^47^. pMD2.G (Addgene plasmid # 12259) and psPAX2(Addgene plasmid # 12260) were gifts from Didier Trono. EX-U0767-Lv105 for overexpressing PD-L1 was purchased from GeneCopoeia. PD-1 (APC, clone MIH4), PD-L1 (APC, clone MIH1), IFNγ (PE-Cy7, clone B27), CD107a (PE, clone H4A3), CD3 (APC-H7, clone SK7), CD4 (PerCP, clone SK3), CD8 (PerCP, clone SK1) and CD8 (BV650, clone SK1) antibodies were purchased from BD Biosciences; IL-2 (BV421, clone MQ1-17H12) was purchased from BioLegend. TNFα (APC, clone MAb11) was purchased from eBioscience.

### Peptides

The following peptides were purchased from Sigma Aldrich: HLA-A*02 restricted M1 58–66 (GILGFVFTL), SSX-2 41–49 (KASEKIFYV), H3 HA 344–354 (RGIFGAIAGFI). They were all assayed to be over 98% of purity.

### Preparation of PBMCs, establishment of CTL lines/clones and cell culture

Blood was taken from median cubital vein of healthy donors by trained phlebotomist (Oxford). Cytotoxic T lymphocyte (CTL) lines and CTL clones were generated from PBMCs (peripheral blood mononuclear cells**)** and stored as previously described^24^. HCV and TAA specific T cell lines and clones generated previously and stored in Oxford T cell clone bank were also used. Cells were cultured in R10 medium (RPMI-1640 supplemented with 10% v/v heat-inactivated foetal calf serum (FCS, Sigma-Aldrich), 2mM L-glutamine (Sigma-Aldrich), 1% v/v (100 U/ml) penicillin streptomycin (Sigma-Aldrich)) or H10 medium (RPMI-1640 supplemented with 10% v/v heat-inactivated human AB serum, 2mM L-glutamine, and 1% v/v (100 U/ml) penicillin streptomycin (Sigma-Aldrich)) with or without 100 U/mL recombinant human IL-2 (Peprotech).

Written informed consent was given from all donors. All methods were performed in accordance with the relevant guidelines and regulations under UK Human Tissue Act 2004.

Ethical approval was obtained from the University of Oxford Tropical Ethics Committee (OXTREC), Xinjiang Tumour Hospital Ethics Committee, Beijing You’an hospital Ethics Committee and London - City & East Research Ethics Committee (REC).

### Pseudotype-viral particle construction and T-cell transduction

A replication incompetent recombinant lentivirus was generated using the three plasmids system by co-transfection of 293TLA cells. A DNA mix composed of 150ng CRISPR/Cas9 inserted lentiviral vector, 100 ng packaging plasmid (psPAX2) and 40 ng envelope plasmid (pMD2.G) per cm^2^ were used. After 48 h, supernatant was collected and filtered by 0.45 μm syringe filter, and concentrated by Lenti-X™ Concentrator (Clontech) according to manufacturer’s instruction. 100,000 cells per well of T cell clones or lines were added to U-bottom 96 well plate and centrifuged at 1,000 g at 32 °C for 2 h with viral particles at desired multiplicity of infection (MOI) in R10 medium. The cells were then cultured in H10 medium for 3 days prior to be checked by flow cytometry.

### PCR, T7E1 assay and Sanger sequencing

Genomic DNA was extracted by Quick Extraction solution (Epicenter). Genomic regions containing the PD-1 target sites (5′-GGC GCC CTG GCC AGT CGT CTG GG-3′) were PCR-amplified using forward primer 5′-CCG ACC CCA CCT ACC TAA GA-3′ and reverse primer 5′- GGG CGG GAT ATG GAA AGA GG-3′ (amplicon size is 452 bp) with KAPA HiFi HotStart ReadyMix PCR Kit (Roche) according to manufacturer’s instruction. The PCR products were then purified using QIAquick PCR Purification Kit (Qiagen). Purified PCR products was then used for T7 endonuclease I (NEB) as described^21^ or cloned with TOPO TA Cloning Kit (Invitrogen) and submitted for Sanger sequencing (Source Biosciences).

### FACS-analysis of T-cells

For each staining, cells were stained with LIVE/DEAD® Fixable Dead Cell Stain Kit (Thermo Scientific) for 20 min first. The surface staining was performed with conjugated antibodies for 20 min. If intracellular staining was required, cells were fixed in Cytofix/Cytoperm^TM^ (BD Biosciences) for 20 min before being stained with conjugated antibodies in 1XPerm/Wash buffer (BD Biosciences) for 20 min. Cells were kept on ice and avoided light throughout the staining procedure to minimize antibody-mediated internalization and degradation of the antibody. Cells were then fixed with Cell Fix solution (BD Biosciences) and acquired on Cyan^TM^ (Beckman Coulter) or Fortessa^TM^ (BD Biosciences) flow cytometer. Cell sorting were performed on FACSAria IIIu (BD Biosciences) with similar procedure except that the cells were stained with 7-AAD (BD Biosciences) instead of LIVE/DEAD® Fixable Dead Cell Stain Kit and kept sterilized.

### T-cell function evaluation assay

Antigen-specific T cells were stimulated with peptide-pulsed autologous B-LCLs in an effector cell to target cell (E:T) ratio of 1:1 or 1:2. For intracellular staining, cells were co-cultured with the presence of anti-CD107a, 0.7 μg/ml monensin (BD Biosciences), 10 μg/ml brefeldin A (BD Biosciences) in R10 medium for 4 h at 37 °C. For supernatant cytokine quantification (Luminex multiplex assay), cells were co-cultured for 24 h and the supernatants were harvested and kept in −80 °C until being analysed using Bio-Plex assay kits (Bio-Rad) on a Luminex machine (Bio-Rad). Negative controls included the supernatant from un-stimulated cells.

### Data analysis

FACS data analysis was performed with Flowjo^TM^ 10 and statistical analysis was conducted using GraphPad Prism 7. *p-*values were calculated using two-tailed unpaired student’s t-test. *p* < 0.05 was regarded as statistically significant.

### Data availability

The datasets generated and analysed during the current study are available from the corresponding author on reasonable request.

## Electronic supplementary material

Supplemental Figures
