# Supplementary material for: Genetic abrogation of immune checkpoints in antigen-specific cytotoxic T-lymphocyte as a potential alternative to blockade immunotherapy
Source: Sci Rep. 2018 Apr 3;8:5549. doi: 10.1038/s41598-018-23803-7 (PMC5882910; doi:10.1038/s41598-018-23803-7)
Supplement: Supplementary file 1 — Supplemental Figures [file 41598_2018_23803_MOESM1_ESM.pdf]

Title:

Genetic abrogation of immune checkpoints in antigen-specific cytotoxic T-lymphocyte as a potential alternative to blockade immunotherapy

Chi Zhang[1], Yanchun Peng[1,2], Philip Hublitz[3], Haokang Zhang[1], Tao Dong[1,2*]

1. MRC Human Immunology Unit, Weatherall Institute of Molecular Medicine, Oxford University, Oxford OX3 9DS, UK
2. CAMS-Oxford Center for Translational Immunology, Nuffield Department of Medicine, Oxford University, OX3 7BN, UK
3. Genome Engineering Department, Weatherall Institute of Molecular Medicine, Oxford University, Oxford OX3 9DS, UK

*Corresponding to: tao.dong@imm.ox.ac.uk

Supplemental Figure 1. Design of sgRNAs to target PD-1.

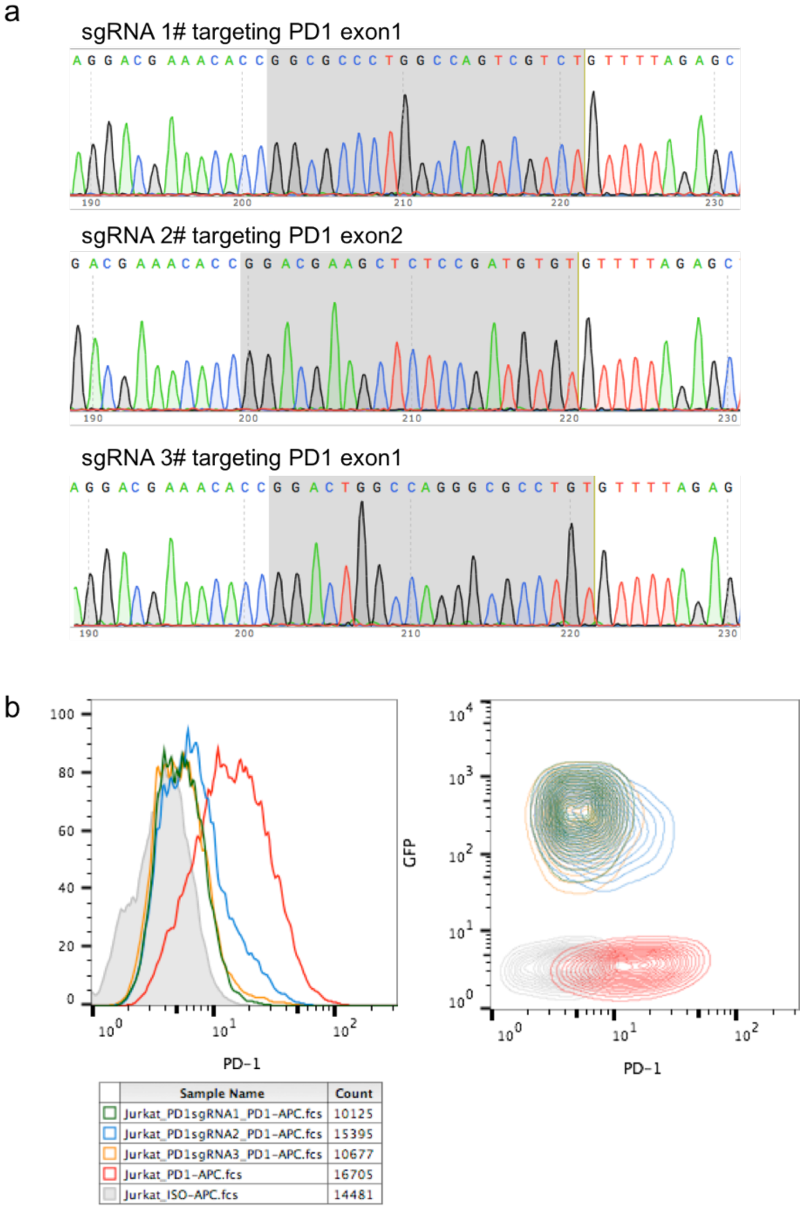

(a) Sequencing results indicate successful insertions of the sgRNAs into pL-CRISPR. SFFV. GFP lentiviral backbone. (b) sgRNA mediated PD-1 disruptions were evaluated by transducing Jurkat T-cells (MOI=10) with the three lentiviruses at same condition. The GFP and PD-1 expressions were checked after three days. sgRNA 1# and 3# (targeting exon1) mediated more efficient PD-1 down-regulations than 2# (targeting exon2). sgRNA 1# was picked to continue in the following experiments.

Supplemental Figure 2. Optimization of lentiviral transduction on Ag-specific CTLs.

a

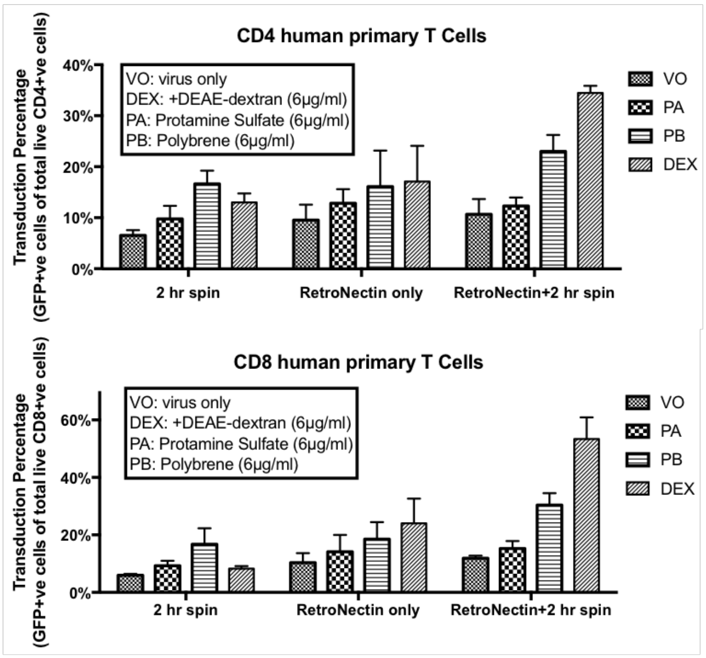

b

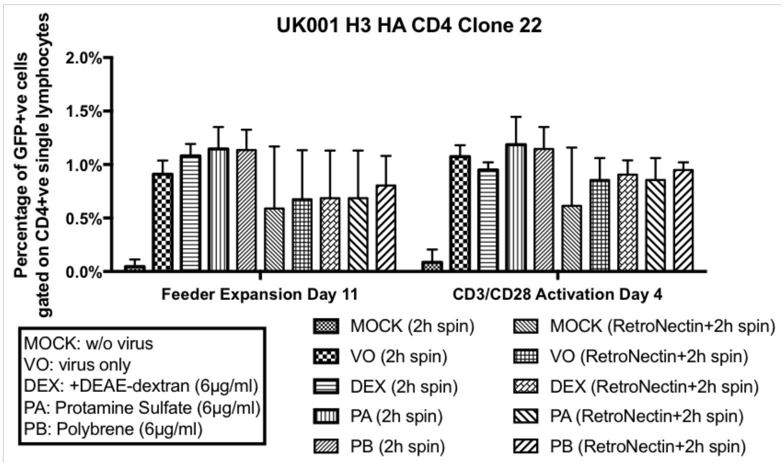

(a) Different kinds of polycations combined with different transduction methods were tested in terms of the facilitating effect of lentiviral transduction on human primary T cells as positive controls. (MOI=10) (b) Clone 22 were either pre-stimulated with anti-CD3/CD28 magnetic beads (beads : cells=1:1) at day 11 post feeder expansion for four days or re-stimulated with feeder cells for 11 days prior to be lentiviral transduced or mock transduced with different combinations of polycations and transduction methods. (MOI=10) After four days, GFP expressions were checked by Cyan flow cytometer. At leave two independent experiments were conducted and we depicted a representative experiment.

Supplemental Figure 3. PD-L1 partially inhibits T-cell degranulation and cytokine secretion.

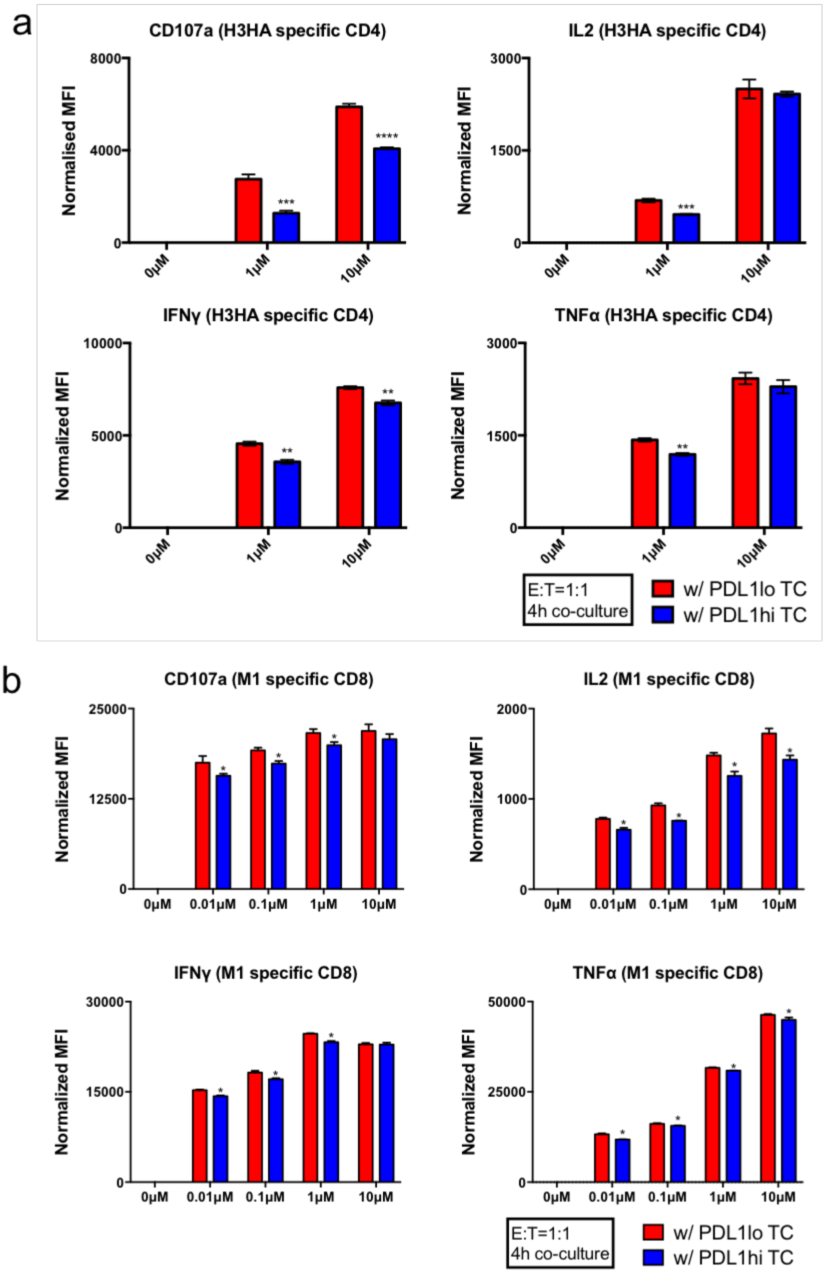

H3 HA specific CD4 T-cell clone 22 (a) and M1 specific CD8 CTL lines (b) were treated with Golgi Stop/Plug and co-cultured with peptide-pulsed PDL1-lo or PDL1-hi (lentiviral overexpressed) HLA-matched BCL (target cells) with corresponding amount of peptide for four hours respectively, and stained for CD107a and IL2, IFNγ, TNFα. Effector cells co-cultured with PDL1-hi target cells showed decreased degranulation (indicated by CD107a staining) and IL2 productions on both CD8 line and CD4 clone. Data shown are mean ± SD of three independent experiments and we depicted a representative out of three experiments yielding similar results. * indicates *p*-value<0.05. ** indicates *p*-value<0.01. *** indicates *p*-value<0.001. **** indicates *p*-value<0.0001. *p*-values were calculated using two-tail unpaired student's *t*-test.

Supplemental Figure 4. PD-1 knock out induces compensatory TIM-3 up-regulation.

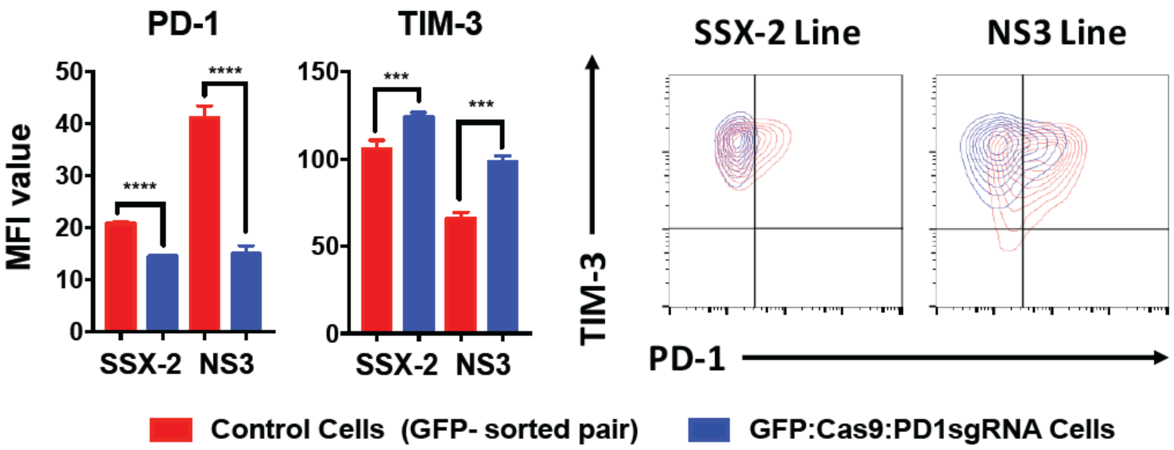

PD-1 and TIM-3 expressions were checked on SSX-2 KV9 lines (SSX-2) and HCV NS3 lines (NS3) 24h post anti-CD3/anti-CD28 stimulation. Data shown are mean ± SD of three independent experiments and we depicted a representative out of three experiments yielding similar results. *** indicates p-value<0.001. **** indicates p-value<0.0001. p-values were calculated using two-tail unpaired student's t-test. Representative FACS overlay plots are shown on the right. Negative populations were determined by FMO controls.
